# Immunosuppressive Mesenchymal Stromal Cells Derived from Human-Induced Pluripotent Stem Cells Induce Human Regulatory T Cells *In Vitro* and *In Vivo*

**DOI:** 10.3389/fimmu.2017.01991

**Published:** 2018-01-25

**Authors:** Clémence Roux, Gaëlle Saviane, Jonathan Pini, Nourhène Belaïd, Gihen Dhib, Christine Voha, Lidia Ibáñez, Antoine Boutin, Nathalie M. Mazure, Abdelilah Wakkach, Claudine Blin-Wakkach, Matthieu Rouleau

**Affiliations:** ^1^LP2M, CNRS-UMR 7370, Faculty of Medicine, Nice, France; ^2^Université Nice-Sophia Antipolis, Nice, France; ^3^Service d’Hématologie Clinique, CHU de Nice, Hôpital de l’Archet, Nice, France; ^4^Pôle d’Odontologie, CHU de Nice, Hôpital Saint-Roch, Nice, France; ^5^Institute for Research on Cancer and Aging of Nice, CNRS-UMR 7284-INSERM U108, Centre Antoine Lacassagne, Nice, France

**Keywords:** induced pluripotent stem cells, mesenchymal stromal cells, human T-cell immunosuppression, regulatory T cells, humanized NSG mouse, tolerance

## Abstract

Despite mesenchymal stromal cells (MSCs) are considered as a promising source of cells to modulate immune functions on cells from innate and adaptive immune systems, their clinical use remains restricted (few number, limited *in vitro* expansion, absence of a full phenotypic characterization, few insights on their *in vivo* fate). Standardized MSCs derived *in vitro* from human-induced pluripotent stem (huIPS) cells, remediating part of these issues, are considered as well as a valuable tool for therapeutic approaches, but their functions remained to be fully characterized. We generated multipotent MSCs derived from huiPS cells (huiPS-MSCs), and focusing on their immunosuppressive activity, we showed that human T-cell activation in coculture with huiPS-MSCs was significantly reduced. We also observed the generation of functional CD4^+^ FoxP3^+^ regulatory T (Treg) cells. Further tested *in vivo* in a model of human T-cell expansion in immune-deficient NSG mice, huiPS-MSCs immunosuppressive activity prevented the circulation and the accumulation of activated human T cells. Intracytoplasmic labeling of cytokines produced by the recovered T cells showed reduced percentages of human-differentiated T cells producing Th1 inflammatory cytokines. By contrast, T cells producing IL-10 and FoxP3^+^-Treg cells, absent in non-treated animals, were detected in huiPS-MSCs treated mice. For the first time, these results highlight the immunosuppressive activity of the huiPS-MSCs on human T-cell stimulation with a concomitant generation of human Treg cells *in vivo*. They may favor the development of new tools and strategies based on the use of huiPS cells and their derivatives for the induction of immune tolerance.

## Introduction

Among the different cells potentially used in regenerative medicine, the mesenchymal stromal cells (MSCs) are viewed as an interesting source of cells, increasingly used in the treatment of various clinical contexts as well as for immunomodulation in conditions linked to auto/allo-immunity (1). These cells are self-renewing, adhere to plastic, express characteristic surface antigens and have mesodermal multilineage differentiation potential *in vitro* (2, 3). MSCs can be obtained from several tissues such as adult bone marrow (BM), adipose tissue and several fetal organs. *Ex vivo* isolated somatic MSCs have been implicated in immune-regulatory functions on cells from both the innate and adaptive immune system. Several secreted factors such as indolamine 2,3-dioxygenase (IDO), transforming growth factor beta (TGF-β), hepatocyte growth factor, and prostaglandin E2 have been shown to mediate their capacity to inhibit T-cell activation [for review, see Ref. (1, 4)]. However, cell-to-cell contact was also shown to be involved in the T cell-inhibitory effect of MSCs, for instance, through targeting cell surface ligands of the B7 super family (5, 6).

Generation of regulatory CD4^+^ T cells through soluble factors produced by MSCs (7) or through interaction between MSCs and monocytes was also shown to mediate immunosuppression of T-cell responses (8). Therefore, MSCs were proposed for cell therapy for treatment of autoimmune related diseases, immunological disorders and acute graft-versus-host disease (9–13), and multiple clinical studies are ongoing (14–19).

Nevertheless, a major restriction for their clinical use is due to the limited *in vitro* expansion of the low quantity of cells that can be collected from adult tissues. Furthermore, their full phenotypic identity *in vivo* remained to be established. Therefore, MSCs derived *in vitro* from human-induced pluripotent stem (huiPS) cells could fulfill some of the specification required to improve MSCs use in therapeutic approaches: well-defined and unlimited number of cells with reproducible functional characteristics.

Several publications reported the generation of pluripotent cell-derived MSCs through embryonic body formation, direct differentiation, or addition of mesenchymal inductors (20–23). These pluripotent cell-derived MSCs express the classical BM-MSC CD44, CD73, CD90, and CD105 markers are capable of *in vitro* differentiation into osteoblasts, adipocytes, and chondrocytes and display some tissue repair activity *in vivo* in mouse models (24). Furthermore, they present an immunosuppressive activity *in vitro* against T cells (25) as well as NK cells (26). The *in vivo* immunosuppressive activity of such cells was so far tested on murine immune cells in different models of immunological disorders such as allergic airways (27), experimental autoimmune encephalomyelitis (25, 28), induced colitis (25), and ischemia (24).

Here, we generated huiPS-MSCs (characterized by the expression of classical markers and their multipotent property) that display *in vitro* an efficient immunosuppressive activity on allogeneic T-cell responses through the induction of regulatory T (Treg) cell differentiation. We further demonstrate that their infusion in humanized NSG mice [human peripheral blood mononuclear cell (PBMC) mouse] induced a decrease in the proportion of human CD4^+^ and CD8^+^ T cells expanding within the mice, along with a switch from a Th1 cytokine profile toward a Treg signature. Our data highlight the promising therapeutic potential of huiPS-MSCs in immune-mediated diseases.

## Materials and Methods

### Cell Culture

All the culture products were provided by ThermoFisher (France) unless mentioned. In this study, the induced pluripotent stem (huiPS) cells were provided by Dr. I. Petit (INSERM U976, Paris) obtained from the reprogramming of human adult fibroblasts (29) or were produced in the laboratory (30). These cells were grown into homogeneous colonies on feeder mouse embryonic fibroblasts (MEFs) treated with mitomycin C (Sigma, France). The culture medium of huiPS cells consisted in 85% DMEM/F12, 15% knockout serum replacement, l-glutamine 100 mM, β-mercaptoethanol 0.1 mM, and bFGF 10 ng/ml (Invitrogen or Peprotech, France). The huiPS cells were passaged one to two times per week by splitting colonies in dissociation buffer (DMEM containing Collagenase type IV 2 mg/ml) without detaching the feeder MEF.

Human iPS-derived mesenchymal stromal cells (huiPS-MSC) were obtained by spontaneous differentiation of huIPS cells. For this, huiPS cells were maintained in huiPS medium without bFGF until the huiPS colonies overgrew. Without passaging them, the differentiating cells were maintained for the next 4–6 days in an “MSC” culture medium containing 30% DMEM, 30% F12, 10% serum FcII (Hyclone, ThermoFisher, France), NEAA 10 mM, Na pyruvate 1 mM, penicillin (1 U/ml)/streptomycin (1 μg/ml), glutamine 1 mM, β-mercaptoethanol 100 μM, ascorbic acid 50 μg/ml (Sigma-Aldrich, France), and huEGF 10 ng/ml. The differentiating cells were then dissociated in PBS 0.05% trypsin–EDTA, and put back in culture in the “MSC” medium. Only few cells collected (<10%) were able to survive and grow (medium was changed 1 or 2 days later to removed dead cells and floating cells). The “MSC” medium was then changed every 3–4 days. Ten to fifteen days later, the cells were passaged (passage 1) and analyzed for the expression of MSC markers (usually 80–90% of cells with MSC phenotype are recovered). These huIPS-MSCs were then maintained in culture and passaged one to two times per week in “MSC” medium. Although the huiPS-MSCs obtained could be maintained up to passage 10, they were used before they reached passage 5.

Human PBMCs were obtained from the EFS (Etablissement Français du Sang) from healthy platelet donors. After separation on a Ficoll gradient, the cells were immediately used or frozen and stored in liquid nitrogen.

### *In Vitro* Differentiation of huiPS-MSCs

Human-induced pluripotent stem-MSCs’ differentiation into adipocytes and chondrocytes was performed using specific differentiation media (StemProAdipocyte medium and StemProChondrogenesis medium, ThermoFisher) according to the manufacturer’s instructions. Osteoblast differentiation was performed in αMEM medium containing 5% Hyclone serum supplemented with ascorbic acid 50 μg/ml, β-glycerophosphate 110 μM, and dexamethasone 0.1 μM. After 14–21 days of culture and fixation, the cells were treated with either Alcian Blue 1% for coloration of chondrocyte matrix, Alizarin Red 2% for osteoblast-derived matrix or Red Oil for presence of lipid droplets as a marker of adipocytes.

### Flow Cytometry Analysis

The cells were labeled for expression of surface markers with fluorescent antibodies in PBS plus 1% FCS and 2 mM EDTA for 30 min at 4°C and were analyzed on a FACS Canto II with the Diva (Becton-Dickinson, France) or the FlowJo (FlowJo, LLC) softwares. The anti-CD90 FITC (clone 5E10), anti-CD105 PeCy7 (clone SN6), and anti-CD3 eFluor450 (clone UCHT1) antibodies were provided by eBioscience SA (France). The anti-CD73 APC (clone AD2), anti-CD4 PeCy7 (clone SK3), and anti-CD8 PerCP-Cy5 (clone SK1) antibodies were provided by Becton-Dickinson.

For intracytoplasmic staining of cytokine expression, human T cells were stimulated for 4 h with Ionomycin (10 μg/ml) and PMA (1 μg/ml) in the presence of Brefeldin A (10 μg/ml) [as already described (31)]. T cells were labeled with the anti-CD4 PeCy7 and anti-CD8 PerCP-Cy5 antibodies, fixed and permeabilized with the Fix/Perm buffer (eBiosciences). They were incubated with antibodies specific (from eBioscience) for the following cytokines: IFNγ (clone 45.B3), TNFα (clone MAb11), and IL-10 (clone Jes3-9D7). A nuclear labeling of fixed and permeabilized human CD4^+^ and CD8^+^ T cells was performed to detect the expression of the FoxP3 transcription factor (APC, clone 259D/C7, Becton-Dickinson). In some experiment, the following antibodies were used CD3 BV510, CD4 PerCP-Cy5, CD127 PeCy7, Neuropilin (Nrp1) BB 515, Helios AF 488, and FoxP3 APC (all from Becton-Dickinson).

### Mixed Lymphocyte Reaction (MLR)

Responder PBMC labeled with 0.4 μM CFSE (ThermoFisher) was stimulated by allogeneic manner in MLR with irradiated (40 Gy) PBMCs from two different donors labeled with 0.4 μM APC-Cell Tracer (eBioscience) and cocultured with or without irradiated (60 Gy) huiPS-MSCs (ratio 1 huiPS-MSC for 10 immune cells) in a u-bottom 96 well plates for 5 days. The cells then were collected and labeled with the anti-CD4 PeCy7 and anti-CD8 PerCP antibodies and then analyzed by flow cytometry. We excluded stimulator cells stained with the APC-Cell Tracer. The proliferation of CD4^+^ and CD8^+^ responder T cells was measured by the dilution of the CFSE marker. In some experiments, the PBMCs were put in the insert of a Transwell culture system (Nunc, 0.45 μm pores), the huiPS-MSC being put in the lower part. A rat anti-human blocking IL-10 antibody (clone JES3-19F1, Becton-Dickinson) and an isotype rat control were used (10 μg/ml) to inhibit the potential role of IL-10 in the immunosuppression.

### The *In Vivo* Model of Human T Cell Expansion in NSG Mice

NOD/SCID/IL2Rγ KO (NSG) mice were purchased from Charles River Laboratory. Animals were maintained in accordance with the general guidelines of the institute. They were injected *ip* with 10 × 10^6^ human PBMC and were treated with or without 1 × 10^6^ huiPS-MSCs by *ip* injection once a week for 3 weeks. In some experiments, mice received only the huiPS-MSCs. Mice were sacrificed between weeks 5 and 7 after PBMC injection. Peritoneal fluid, blood, and spleen were collected for flow cytometry analysis. Approval for the use of mice in this study was obtained from our local Institutional Ethic Committee for Laboratory Animals (CIEPAL-Azur, NCE/2013-102).

### ELISA

We determined by ELISA the concentrations of human IL-1a, IL-6, IL-8, IL-2, and IFNγ (Development kit, Peprotech) in MLR culture media according to the manufacturer’s instructions.

### RNA Extraction and Quantitative Real-time PCR

Total RNA was extracted and reverse transcribed (SuperScript II Reverse Transcriptase, Invitrogen), and real-time RT-PCR was performed on an StepOnePlus Fast real-time PCR system (SybR Green, Applied Biosystems) on triplicates as described. Results were normalized to the different housekeeping genes (*ACTIN, GAPDH*, and *UBIQUITIN*) on the same plate. Differences in gene expression were calculated using the 2^−ΔΔCt^ method.

### Statistical Analysis

Results are presented as mean ± SD. The level of statistical significance was determined by the unpaired two-sample Student’s *t*-test. *p* Values <0.05 were considered statistically significant.

## Results

### Characterization of huiPS-MSCs: Phenotype and *In Vitro* Multipotency

**Figure 1:**
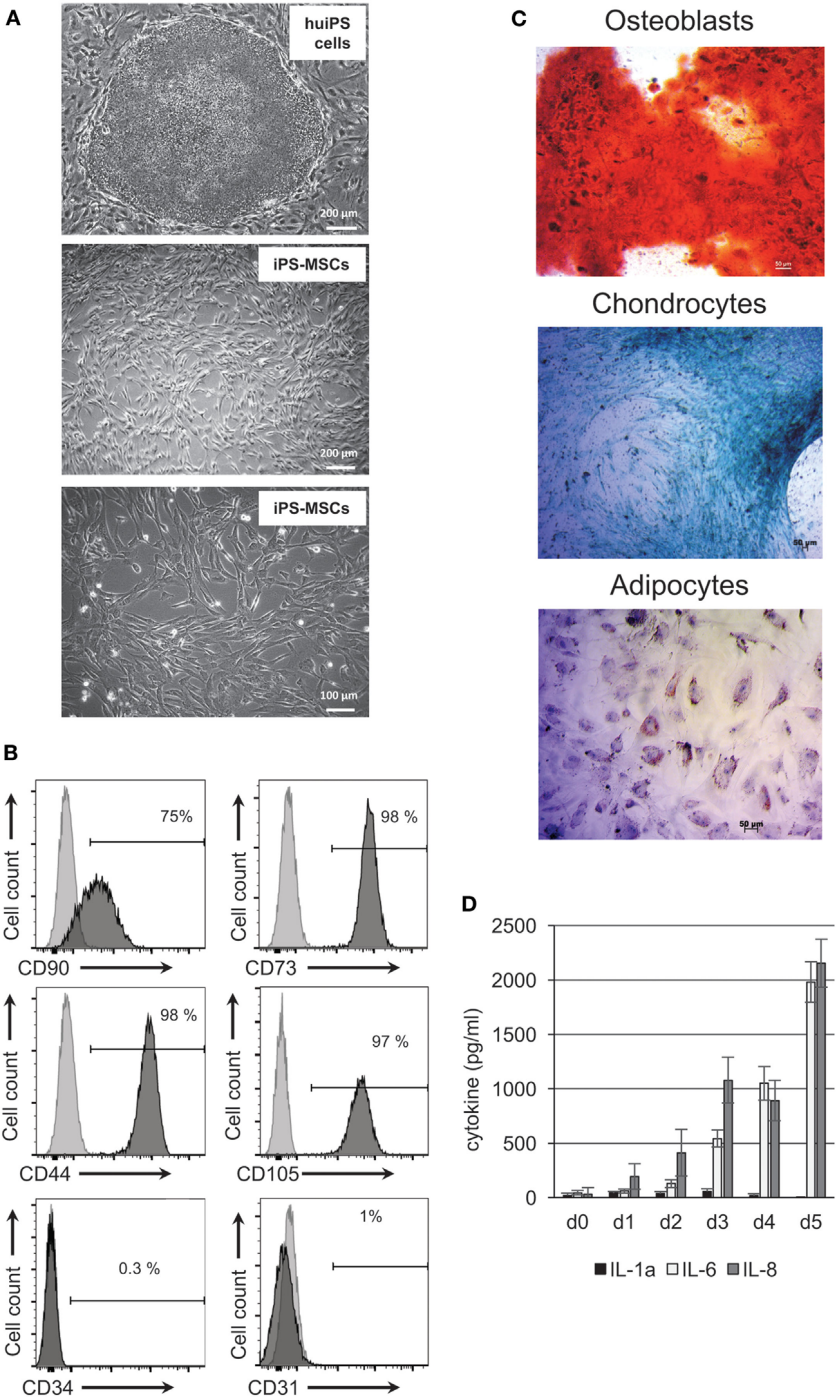
Characterization of human-induced pluripotent stem (huiPS) cells differentiated in huiPS-mesenchymal stromal cells (MSCs). **(A)** The picture in the upper panel shows undifferentiated huiPS cells in culture on mouse embryonic fibroblast feeder cells (bar = 200 μm); the two bottom pictures represent huiPS-MSCs showing typical shapes and obtained after two passages in differentiated medium (bars = 200 and 100 μm, respectively). **(B)** Flow cytometry analysis of huiPS-MSCs showing the typical expression of CD90, CD73, CD105, and CD44 and the absence of expression of CD31 and CD34. **(C)** Multipotency of huiPS-MSCs was assayed after culture in specific medium for 14 days for the adipocyte and chondrogenic differentiation or for 21 days for the osteogenic differentiation. Specific extracellular matrix components were colored with Alzarin Red (for osteoblast) and Alcian Blue for chondrocytes, while adipocytes were detected with Oil Red coloration of lipid droplets. **(D)** Histogram bars representing the level of IL-1α, IL-6, and IL-8 cytokines produced *in vitro* by huiPS-MSCs, as detected by ELISA, after 1–5 days in culture (mean ± SD of values from three independent experiments expressed as picograms per milliliter).

Because previous differentiation protocols of MSCs from pluripotent cells involve many steps ranging from embryonic body formation to cell sorting or both (20, 22, 32–36), we set up a simple two-step protocol that would generate rapidly mesodermal-derived cells (Figure 1A). Cultured human iPS cells were kept in absence of bFGF for 7 days, followed by a change for an ectodermal/mesodermal medium (37) for the next 2 weeks. Eighty to ninety percent (at passage 1) and 100% (at passage 2) of the recovered cells (Figure 1B) express surface antigens (Ags) known to be expressed by tissue-derived MSCs. They were positive for CD44, CD73, CD90, CD105, and HLA-ABC Ags but negative for the endothelial CD31 marker, the hematopoietic and immune related CD34, CD45 markers, HLA-DR antigens, and CD80 and CD86 co-stimulatory molecules (Figure 1B; Figure S1 in Supplementary Material). Three different human iPS cell lines, prepared from different donors and from different tissues [skin fibroblast (29, 38) or myoblasts (30)] behaved identically, confirming that this protocol is applicable for multiple human iPS cells.

In addition, the cells recovered and further kept in culture were capable of differentiation into the classical mesenchymal-derived cells (osteoblasts, chondrocytes, and adipocytes) (Figure 1C) when cultured with appropriate differentiation media, suggesting that the huiPS-MSC population contained multipotent cells and correspond to *bona fide* MSCs. Finally, the huiPS-MSCs we have generated secreted high and sustained amount of IL-6 and IL-8 cytokine/chemokine but low amount IL-1α (as tested by ELISA) (Figure 1D), a cytokine profile shared by tissue-derived MSCs and associated with their role in tissue repair (39, 40). Altogether, these results highlight that the huiPS-MSCs generated with our protocol shared strong similarities with *in vitro* maintained MSCs derived from adult tissues (2, 3).

### *In Vitro* Immunosuppressive Activity of huIPS-MSCs on Activated T Lymphocytes

**Figure 2:**
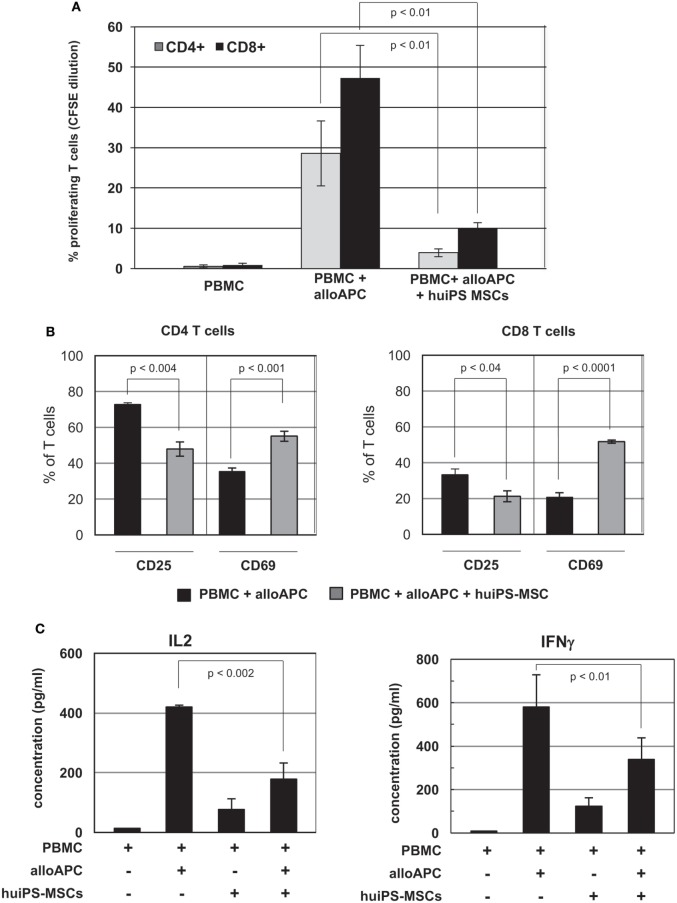
*In vitro* immunosuppressive activity of human-induced pluripotent stem (huiPS)-mesenchymal stromal cells (MSCs) on human T lymphocytes. **(A)** *In vitro* immunosuppressive activity of huiPS-MSCs on human CFSE-labeled peripheral blood mononuclear cell (PBMC) stimulated for 5 days with irradiated allogenic PBMC (as alloAPC) in the presence or absence of huiPS-MSCs. The percentage of proliferating CD4^+^ and CD8^+^ T cells (assessed by CFSE dilution) was then determined by flow cytometry. The graph represents the mean ± SD of values from five independent experiments, with the corresponding *p* values. **(B)** The proportion of CD4^+^ (left panel) and CD8^+^ (right panel) T cells expressing CD25 and CD69 was determined by flow cytometry. The histogram represents the mean % ± SD with the corresponding *p* values calculated from three independent experiments. **(C)** Analysis by ELISA of IL-2 and IFNγ cytokines (pg/ml) at day 4 in cultures of allogenic stimulated PBMC in the presence or absence of huiPS-MSCs. The graphs represent the mean ± SD of values from three independent experiments, with the corresponding *p* values.

Besides their multipotent characteristics, tissue MSCs display *in vitro* immunosuppressive activities. To test the immunosuppressive properties of the huiPS-MSCs, we analyzed their action on the proliferation of human T lymphocytes stimulated in an allogeneic manner (Figure 2A). The stimulation of PBMCs in MLR with allogeneic antigen-presenting cells (PBMC from a secondary donor—in here named alloAPC) resulted in CD4^+^ and CD8^+^ T-cell proliferation, which was significantly reduced in coculture with huiPS-MSCs. We further observed a significant reduction in the % of CD25-expressing CD4^+^ and CD8^+^ T cells, indicating a diminished proportion of activated T cells in coculture with huiPS-MSCs (Figure 2B). Another activation marker (CD69) was on the contrary expressed on a higher % of CD4^+^ and CD8^+^ T cells (Figure 2B). Known to be an early activation marker, its maintained expression might indicate that the immunosuppression required to be effective the early activation of the T cells. It is also expressed on memory T cells, which would suggest that the remaining T cells in the coculture might acquire such a “memory” phenotype. We also analyzed by RT-qPCR the relative expression of mRNAs coding for cytokines as well as some surface receptors involved in such immune reactivities (Figure S2 in Supplementary Material). As expected, gene expression signature of activated T cell was clearly reversed upon exposure with huiPS-MSCs. Indeed, compared with the RNA expression by huiPS-MSCs alone or in coculture with unactivated T cells, the relative expressions of activated T-cell cytokines (IL-2, IFNγ, TNFα, and TNFβ) were reduced in the MLRs in the presence of huiPS-MSCs (Figure S2A in Supplementary Material). Measured by ELISA (Figure 2C), the IL-2 and IFNγ production was indeed reduced in the cocultures with huiPS-MSCs confirming at the protein level the lower activation of T lymphocytes observed in such conditions. Furthermore, RNA expression of other genes coding for cytokines were affected (Figure S2A in Supplementary Material): we observed an increased expression of inflammatory IL-1α and IL-1β, IL-6 (cytokines that were shown to sustain the immunosuppressive activity of MSCs), as well as TGF-β and LIF known for their immunosuppressive functions. The mRNA coding for the cytokine IL-10 was also overexpressed. However, its immunosuppressive function appeared to be not involved in the T-cell immunosuppression. Indeed, using anti-IL-10-blocking antibodies during the initial MLR in the presence of huiPS-MSCs did not affect the inhibition of the T-cell proliferation (Figure S3A in Supplementary Material). Tested in transwell assay, we observed that the level of inhibition of the CD4^+^ T-cell proliferation was partially reduced (Figure S3B in Supplementary Material). This indicated that both cellular contacts between activated T cells and huiPS-MSCs, and soluble factors were involved in the immunosuppressive function of the huiPS-MSCs. On the contrary, the proliferation of the CD8^+^ T-cell population appeared to be less inhibited in the transwell assay, suggesting that their immunosuppression required a direct contact between T cells and huiPS-MSCs. The relative RNA expression of surface molecules involved in the T-cell activation and their polarization into effector cells such as OX40 and its ligand (OX40L), as well as CD47 were diminished, while on the contrary, those of LAG3 and CTLA4, potentially involved in immunosuppressive functions were increased in the MLR in the presence of huiPS-MSCs (Figure S2B in Supplementary Material). Altogether, these observations might indicate a change in the polarization of the T-cell populations activated in the presence of huiPS-MSCs.

### huiPS-MSCs Induced a Switch in T Cell Polarization

**Figure 3:**
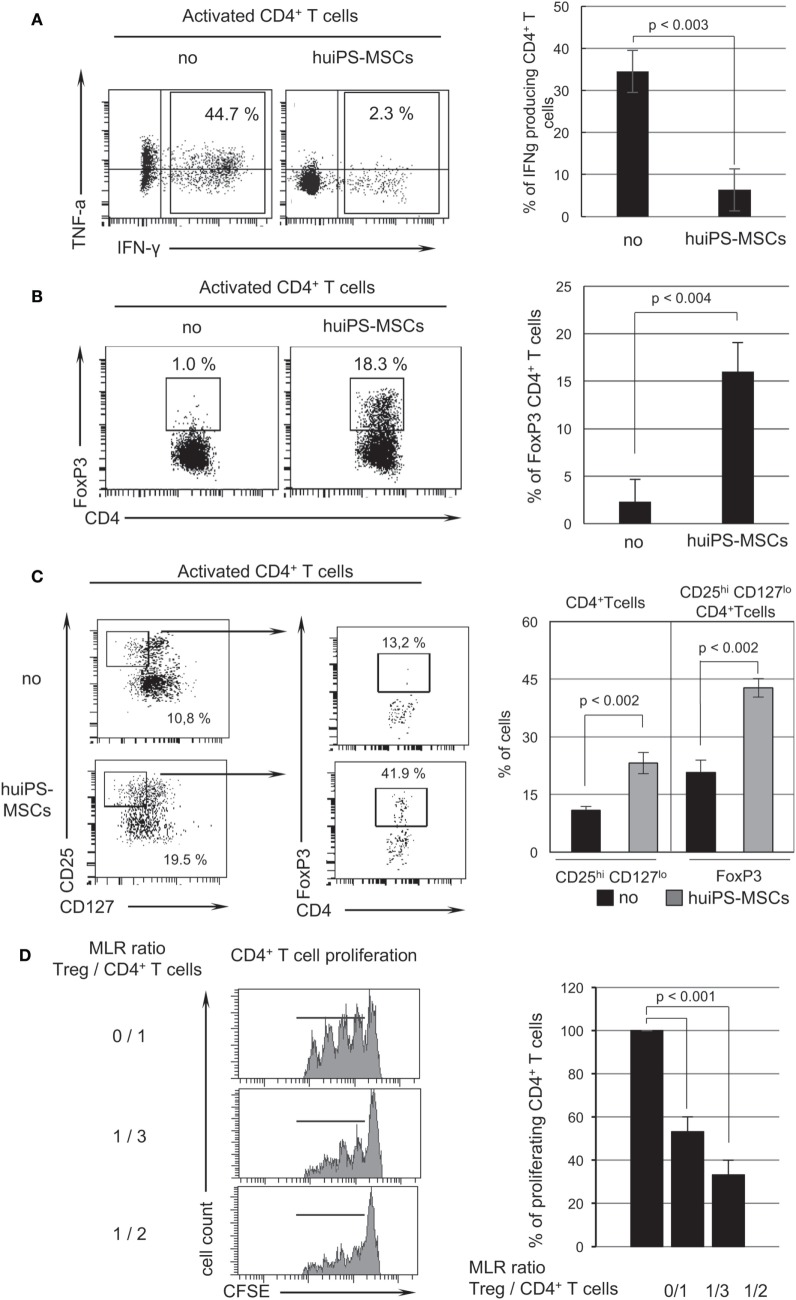
Switch in T-cell effector function induced by human-induced pluripotent stem (huiPS)-mesenchymal stromal cells (MSCs). **(A)**. Analysis by flow cytometry of intracytoplasmic production of Th1 cytokines (IFNγ and TNFα) performed on CD4^+^ T lymphocytes after allogenic stimulation without (no) or with huiPS-MSCs cocultures. The histogram represents the % of CD4^+^ T cells expressing INFγ (mean % ± SD and *p* value calculated from three independent experiments). **(B)** Detection of CD4^+^ FoxP3^+^ regulatory T (Treg) cells after mixed lymphocyte reaction (MLR). The dot plots show the % of FoxP3-expressing CD4^+^ T cells determined by flow cytometry after specific intranuclear staining of FoxP3 performed on CD4^+^ T lymphocytes after allogenic stimulation without (no) or with huiPS-MSCs cocultures. The histogram represents the mean % ± SD with the corresponding *p* values calculated from three independent experiments. **(C)** The proportion of CD4^+^ T cells expressing high level of CD25 (CD25^hi^) and no or low level of CD127 (CD127^lo^) was determined by flow cytometry. Further gated on this population, the level of FoxP3 expression was analyzed. The histogram represents the mean % ± SD with the corresponding *p* values calculated from two independent experiments. **(D)** Treg cells obtained in the MLR in the presence of huiPS-MSCs are immunosuppressive *in vitro*. Mix lymphocyte reactions (with different cell ratios) were realized between activated CFSE-labeled CD4^+^ T cells and the CD4^+^ Treg cell containing population obtained from previous coculture of T cells with huiPS-MSCs. The left panel shows a representative graph indicating the level of CFSE dilution (i.e., proliferating CD4^+^ responding T cells) at three different ratios after 4 days in culture and analyzed by flow cytometry. The right panel displays the proportion of proliferating CD4^+^ T cells expressed in percentage of proliferation of CD4^+^ T cells in the absence of the Treg cell containing population. The histogram represents the mean % ± SD with the corresponding *p* values between ratio 0/1 and either ratio 1/3 or 1/2 calculated from three independent experiments.

Using intracytoplasmic cytokine detection by flow cytometry in activated T lymphocytes, we observed a dramatic reduction in the proportion of CD4^+^ T lymphocytes producing IFNγ and TNFα corresponding to Th1 cells in MLR with huiPS-MSCs (Figure 3A). Furthermore, we detected the presence of CD4^+^ Treg cells among the T populations recovered from the cocultures. As shown in Figure 3B, the MLR without huiPS-MSCs generated only a small amount of FoxP3^+^ CD4^+^ T cells, while in the presence of huiPS-MSCs this percentage strongly increased up to 16%. FoxP3 being susceptible to reflect human T-cell activation, we confirmed the presence of CD4^+^ Treg cells since we observed an increased detection of a population of CD4^+^ T cells expressing a high level of the CD25 and a low level of CD127 markers (Figure 3C). This CD4^+^ CD25^hi^ CD127^lo^ T-cell population further contained a higher proportion of FoxP3 cells (Figure 3C). To better define the phenotypic characteristic of the Treg cell population induced in the presence of huiPS-MSCs, we showed that the neuropilin1 (Nrp1) surface marker was not particularly expressed by such Treg cells and that the Ikaros family member Helios was clearly highly expressed in the whole CD4^+^ T cells population (reflecting a global activation) (Figure S4 in Supplementary Material). Finally, this CD4^+^ T-cell population recovered from the cultures in the presence of the huiPS-MSCs was then assayed in a secondary MLR. As shown in Figure 3D, this CD4^+^ T-cell population containing Treg cells was very efficient to inhibit CD4^+^ T-cell proliferation. Indeed, we observed 50 and 70% inhibition of cell proliferation when the ratios of Treg cells over CD4^+^ T cells were 1 for 3 and 1 for 2, respectively. We thus demonstrated that the FoxP3^+^ CD4^+^ T cells generated during the coculture with the huiPS-MSCs are immunosuppressive CD4^+^ Treg cells.

Altogether, these results highlight the immunosuppressive activity *in vitro* of the huiPS-MSCs on T-cell stimulation that induces a switch in T-cell cytokine polarization and the generation of Treg cells.

### *In Vivo* Suppressive Activity of huiPS-MSCs

**Figure 4:**
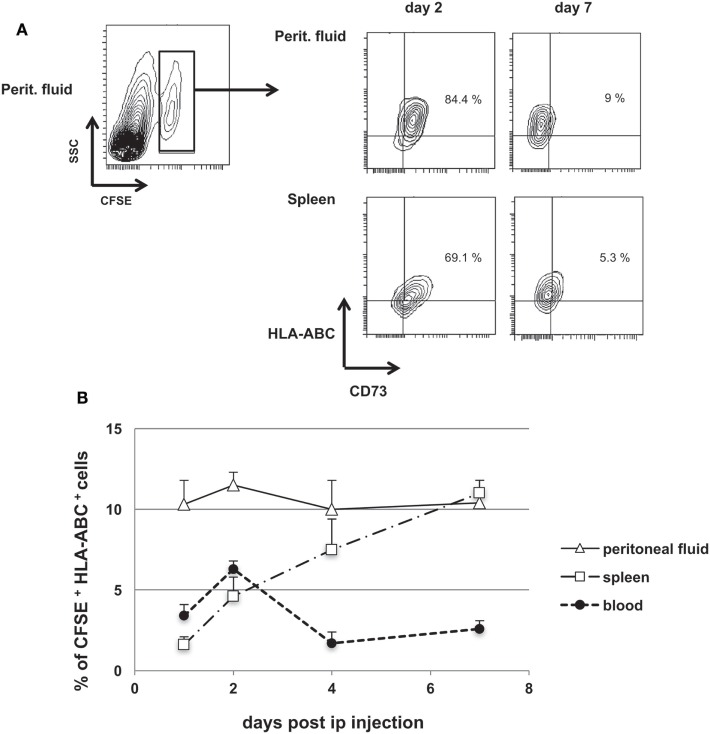
Human-induced pluripotent stem (huiPS)-mesenchymal stromal cells (MSCs) injected in NSG mice remained detectable for 7 days. **(A)** CFSE-labeled huiPS-MSCs were injected *ip* (1 × 10^6^ cells) and were detected within NSG mouse peritoneal fluid and among splenocytes through their CFSE label and their expression of HLA-ABC and CD73 by flow cytometry (% of positive cells are given). Days 2 and 7 after injection are represented. Note that CD73 expression progressively decreased *in vivo*. **(B)** CFSE^+^ HLA-ABC^+^ huiPS-MSCs were detected up to 7 days after injection in the peritoneal fluid, blood, and the spleen of NSG mice. The graph represents the percentage of huiPS-MSCs among all cells recovered from the different mouse tissues at different days after *ip* injection. The graph displays the mean % ± SD of triplicate determinations from two mice sacrificed at the indicated days and is representative of two independent experiments.

We further tested the immunosuppressive activity of huiPS-MSCs in a model of human T-cell expansion in immune-deficient NSG mice (41). First, we determined whether *ip*-injected huiPS-MSCs could be detected in different compartments in NSG mice. HLA-ABC^+^ huiPS-MSCs were labeled with CFSE and injected *ip* in NSG mice. Despite a lower level of expression of CD73 on recovered cells, we showed that CFSE^+^ HLA-ABC^+^ huiPS-MSCs could be detected up to 7 days after injection not only within the peritoneal cavity but also among the circulating cells and splenocytes (Figures 4A,B). This indicated that the injected huiPS-MSCs remained viable for at least 7 days within these mice and were able to circulate at least up to the spleen.

**Figure 5:**
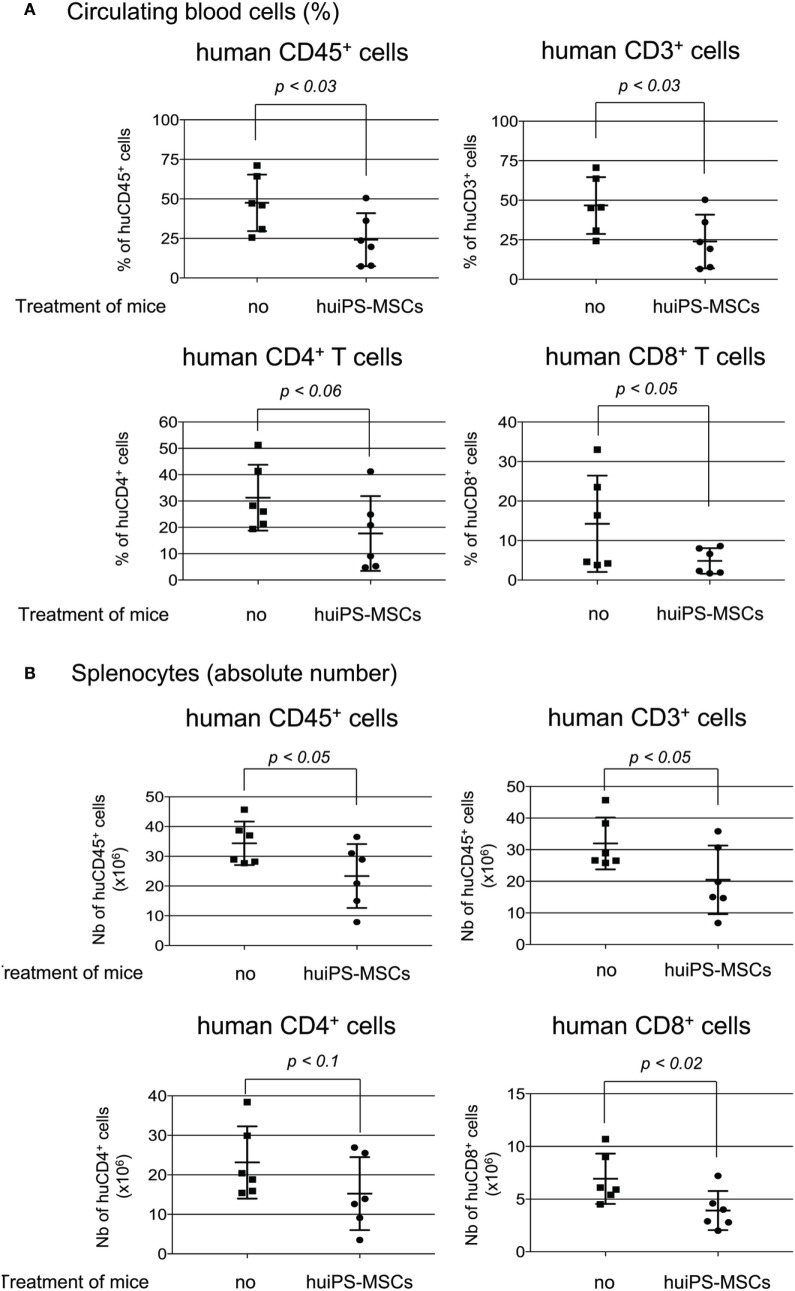
*In vivo* immunosuppressive role of human-induced pluripotent stem (huiPS)-mesenchymal stromal cells (MSCs) infusion in a model of human T-cell expansion in NSG mice. NSG mice injected with human peripheral blood mononuclear cell (PBMC) were treated or not (no) with three infusions of 1 × 10^6^ huiPS-MSCs (at 1-week intervals), and human cells were analyzed by flow cytometry 5–7 weeks after PBMC injection. **(A)** % of Human CD45-, CD3-, CD4-, and CD8-positive-circulating cells recovered from the blood. **(B)** Absolute number of human CD45-, CD3-, CD4-, and CD8-positive cells recovered from spleens. Six mice per group were analyzed. The graphs display the value for each mouse (squares for not injected and rounds for huiPS-MSCs-injected mice) and their mean (horizontal bar) ± SD. The *p* values between not injected and huiPS-MSCs-injected mice are indicated for each panel. Data are representative of three independent experiments.

In a second step, we tested their impact *in vivo* on the expansion of human T cells. NSG mice were injected with human PBMC, were then treated or not with three infusions of huiPS-MSCs (through *ip* injection at 1-week intervals), and were sacrificed between weeks 5 and 7. After sacrifice, cells collected from the peritoneal cavity, those circulating in the blood and those present in the spleen were analyzed by FACS analysis (not shown and Figure 5) on the basis of expression of the human CD45 Ag. Within the peritoneal cavity, human CD45^+^ cells represented about 65% of total cells recovered from control or huiPS-MSCs-treated mice; more than 95% of them were CD3^+^ T cells (not shown). This indicated that among the injected PBMC, T cells were the main human cell population able to expand and colonize the mice. Accordingly, we observed in the blood and spleen that more than 90% of human CD45^+^ cells were CD3^+^ T cells with expected proportion of CD4^+^ and CD8^+^ cells, indicating a similar rate of expansion. But when mice were treated with huiPS-MSCs, the percentage of circulating human T cells was significantly reduced about 1.8-fold (Figure 5A) leading to a 1.6-fold reduced accumulation of total T cells within the spleen (Figure 5B). Interestingly, the proportion of both CD4^+^ and CD8^+^ T cells was changed, the CD8^+^ T cells being significantly more affected by the huiPS-MSCs treatments (Figure 5B).

**Figure 6:**
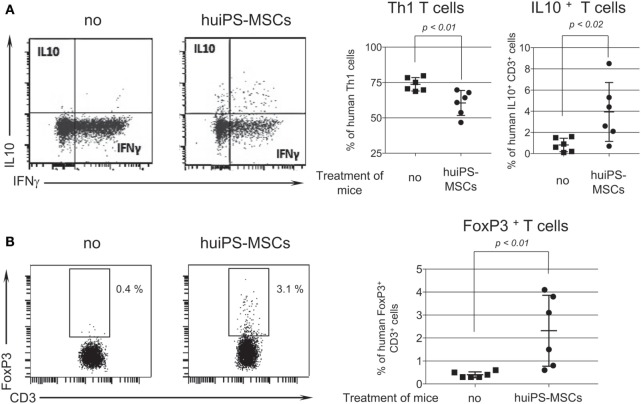
Increased proportion of regulatory T cells after human-induced pluripotent stem (huiPS)-mesenchymal stromal cells (MSCs) infusion in the NSG mouse model of human T-cell expansion. **(A)** The left panels display a representative intracytoplasmic labeling of human T cells recovered from spleens of peripheral blood mononuclear cell (PBMC)-humanized NSG injected (huiPS-MSCs) or not (no) with huiPS-MSCs for the detection of IFN-γ- and IL-10-producing T cells. The right panels represent the percentage of Th1 cells and IL-10-producing T cells in mice injected or not with huiPS-MSCs. The graphs display the % of positive T cells for each mouse (squares for not injected and rounds for huiPS-MSCs injected mice), the mean (horizontal bar) ± SD. The *p* value between not injected and huiPS-MSCs-injected mice is indicated for both panel. Six mice per group were analyzed. **(B)** The left panels display a representative intracytoplasmic labeling of human T cells recovered from spleens of PBMC-humanized NSG injected or not with huiPS-MSCs for the detection of FoxP3-expressing T cells. The right graphs display the % of FoxP3-positive T cells for each mouse (squares for not injected and rounds for huiPS-MSCs injected mice, *n* equal 6 per group), the mean (horizontal bar) ± SD. The *p* value between not injected and huiPS-MSCs-injected mice is indicated. Data are representative of two independent experiments.

Intracytoplasmic labeling of cytokines produced by the human T cells recovered from the spleen was performed. We showed that untreated mice displayed high percentages of human inflammatory IFNγ^+^ TNFα^+^ Th1 cells, while little or none produced the anti-inflammatory IL-10 cytokine (Figure 6A). By contrast, in mice treated with the huiPS-MSCs, the proportion of Th1 cells was substantially reduced, while the one of T cells producing IL-10 was increased more than sixfolds. In parallel, FoxP3^+^ CD3^+^ Treg cells were absent in non-treated animals whereas they were systematically detected in huiPS-MSCs injected mice (Figure 6B).

Altogether, these data demonstrated that the huiPS-MSCs were able to limit the human T-cell expansion *in vivo*, along with a reduced Th1 inflammatory cytokine profile, the presence of IL-10-producing T cells and the generation of FoxP3^+^ Treg cells.

## Discussion

Mesenchymal stromal cell clinical use, through many therapeutic protocols, has proven its safety and efficacy in the treatment of various degenerative diseases and tissue injuries. Since the pioneering work by Takahashi et al. on the derivation of induced pluripotent stem cells (42, 43), tremendous progresses enables to imagine the future safe clinical applications of such cells in regenerative medicine.

In this study, huiPS cells were used to efficiently generate stromal mesenchymal cells (huiPS-MSCs) with a simple spontaneous differentiation method. As other studies evaluating the properties of MSCs derived from pluripotent cells (21), we confirmed that the cells we obtained exhibit the morphologic, phenotypic, and immunomodulatory characteristics assigned to adult tissues—or cord blood-derived MSCs (4). They fulfill the defined standards attributed to *in vitro*-expanded MSCs derived from BM with the lack of expression of specific hematopoietic and endothelial cell markers, and as expected, they express the MSC-identifying markers CD73, CD90, and CD105 (3). As their *ex vivo* counterpart, the huiPS-MSC population we generated is able to give rise, in appropriate culture conditions, to osteoblasts, chondrocytes, and adipocytes revealing similar multipotent property. Furthermore, they produced IL-6 and IL-8, cytokines known to be associated with MSC tissue repair potential (39, 40).

Among the different approaches used to differentiate pluripotent cells into MSCs, either embryonic bodies formation or the use of flow cytometry cell sorting might complicate the large-scale production of such cells for industrial or clinical applications. The protocol we set up allows to generate huiPS-MSCs in 2 weeks from confluent iPS cell cultures. This simple protocol, fast and not expensive, revealed to be efficient and reproducible. Nevertheless, the cellular and molecular mechanisms involved in the *in vitro* differentiation of MSCs from pluripotent stem cells in our hand and in many of the published studies remained to be characterized. Some insights were recently provided indicating that MSCs could be derived from hES cells *via* a trophoblast-like intermediate state (25) or increased through the inhibition of the IKK/NF-κB signaling pathway (23). Such studies provide new tools not only to generate efficiently MSCs *in vitro* but also for a better understanding of the *in vivo* origin of MSC populations.

We also analyzed *in vitro* some of the mechanisms of immunosuppression exhibited by the huiPS-MSCs cells we have generated. Many *in vitro* and *in vivo* studies reported the potent immune modulating functions of tissue-derived MSCs through action on different types of immune cells, activated through variable means (1, 4). Concentrating on human T cells in our study, stimulation of PBMC by MLR in coculture with the huiPS-MSCs resulted in a dramatically decreased proliferation of both CD4^+^ and CD8^+^ T lymphocytes and a concurrent decrease in IL-2 and IFNγ production, both cytokines associated with inflammatory activation of T cells.

The transcriptomic analysis we performed gave some insight into the potential mechanisms involved in the immunosuppression. While we observed a contradictory increased expression of IL-1α, IL-1β, and IL-6, considered as “inflammatory” cytokines, those were shown to be necessary sustain the immunosuppressive activity of MSCs (1) and could therefore participate to the overall immunosuppression. We also noticed a diminished expression of co-stimulatory molecules involved in the T-cell activation and in their polarization into effector cells such as OX40L (44) and CD47 (45). On the contrary, those of LAG3 (46, 47) and CTLA4 (48, 49), both described for their potent immunosuppressive functions on T cells, were increased in the MLR in the presence of huiPS-MSCs. Likewise, the higher RNA expression of IL-10, TGF-β, and LIF, well-known strong immunosuppressive cytokines (1, 50), strengthens the immunosuppressive action on T cells by the huiPS-MSCs. To be noticed, even if the level of RNA expression for PD-L1 (B7H1, CD274), one other well-known immunosuppressive molecule appeared reduced upon exposure of activated PBMCs to huiPS-MSCs (compared with activated PBMCs), the involvement of this pathway is clearly engaged (not reported in here). Altogether, our data point out the package of multiple mechanisms contributing to inhibit the T-cell immune system used by the huiPS-MSCs similarly to tissue somatic MSCs.

Finally, among the molecular pathways able to impact effector T cells, the huiPS-MSCs we have generated were able to induce *in vitro* the differentiation of functional CD4^+^ Treg cells expressing FoxP3 as well as CD25 at high level and not expressing CD127 (51), at the expense of IFNγ^+^ TNFα^+^ inflammatory T cells. Treg cells were shown to depend on TGF-β signaling for maintenance of their immunosuppressive function (52). Such mechanism may be involved in our hand as the huiPS-MSCs produced a very high amount of TGF-β (not shown). It remains to determine whether such a Treg cell population is indeed induced *in vitro* or simply expanded from natural Treg present within the PBMC used (53). The expression of the Neuropilin1 or the Helios markers could be used to discriminate between natural and induced Treg cells (54–57). However, this remains controversial (57–60). The population we generated did not expressed Neuropilin1, consistent with an induced phenotype. Regarding Helios expression, it was highly expressed on the Treg cells tested. But Helios has also been described to be highly expressed on induced Treg cells and is a marker of T-cell activation and proliferation (61, 62). Nevertheless, the presence of cytokines such as IL-2 (which level is inhibited but not abrogated), TGFβ, and IL-6 in the MLR we have performed with huiPS-MSCs suggest that the Treg cells obtained could be induced *in vitro* (63).

Interestingly, because of these different characteristics, one might consider the clinical use of huiPS-MSCs in strategies aiming at inducing immune tolerance toward other human iPS cell type derivatives. Indeed, the clinical use of autologous iPS-derived cells might be compromised by the overall genetic instability generated during the reprogramming of epigenetic defined somatic cells. Allogenic, genetically stable, iPS cell line banks could be the clinical alternative provided that the challenge of immune rejection of transplanted allogenic cells would be resolved. As discussed recently by Liu et al. (64), some proposed strategies that could be considered, involve known tolerogenic pathways through forced expression of CTLA4-ig and PD-L1 by iPS-derived cells. Because they are able to generate Treg cells and probably innately use CTLA4/CD28 as well as PD-L1/PD1 axes on T cells to block their reactivity [as does the tissue MSCs—(1, 4)], huiPS-MSCs could be included within the arsenal of tools used to promote immune tolerance as an associated cell type proposed along with the therapeutic transplanted iPS-derived cells.

To the best of our knowledge, this study is the first evaluating the huiPS-MSCs immune-regulatory properties on human T-cell responses *in vivo* through the potential generation of Treg cells. The model of humanized NSG mouse allows to evaluate the state of activation of human T cells recovered from different organs (peritoneal fluid, blood, and spleen) after treatment with the huiPS-MSCs. Indeed, injection of human PBMC in such immunocompromised mice led to the expansion, circulation, and accumulation (within the spleen) of human activated CD3^+^ T cells, other CD45^+^ cells (such as B lymphocytes, NK cells, or monocytes) being barely detected within the different tissues. Furthermore, NSG mice were not irradiated before human cell injections [as done in many NSG models (65–67)] to avoid possible repair mechanisms of huiPS-MSCs on irradiated tissues that could interfere with their immune suppressive action on T cells. Finally, the huiPS-MSCs were injected *ip* instead of *iv* to prevent possible pulmonary embolization known to lead to massive secretion into the blood of immune modulating factors such as TSG6 (68). This *ip* mode of injection did not confine the huiPS-MSCs to the peritoneal cavity since we were able to detect them over 7 days in the blood stream as well as in the spleen. These results indicate that huiPS-MSCs were able to migrate to different area, colonized as well by human activated T cells, where they might exert their immunosuppressive functions.

Using this model setup, we confirmed *in vivo* the inhibitory action of huiPS-MSCs cells on the proliferation of T cells since we observed a decreased expansion of the CD3^+^ T-cell population in huiPS-MSC-injected mice. Even if this impact appears to be more pronounced on the CD8^+^ than CD4^+^ T-cell populations, both T-cell populations were affected.

Interestingly, in the case of allogenic stem cells transplantation, MSC-treated patients presented a higher level of IL-2 in their serum and a higher Th2 cytokine (IL4) profile at the expense of the Th1 cytokine (IFNγ) profile (18). Our results indicate that treatment with the huiPS-MSCs induces a switch from a Th1 signature toward a regulatory signature (with increased IL-10 production) and the generation of FoxP3^+^ Treg cells. Nevertheless, not all clinical studies using MSC infusion in human reported such clear shifts in T-cell responses, possibly due to different clinical settings. Our results highlight that the induction of Treg cells may be a substantial mechanism by which huiPS-MSCs and probably adult MSCs exert their function *in vivo*. Interestingly, Gregoire-Gauthier et al. (69) reported that cord blood-derived MSCs were able to delayed clinical sign of acute GVHD in irradiated NSG mice through healing process and immunomodulation not related to Treg cells. This supports that the function of MSCs may also differ depending on the experimental settings and this might explain some of the contradictory results of clinical studies.

Co-transplantation of MSCs during allogeneic hematopoietic stem cell transplantation has been explored to enhance engraftment and decrease the risk of graft-versus-host disease (GVHD). However, although several preclinical and clinical studies with MSCs have been conducted, the results have been mixed and the efficacy of MSCs in a transplantation setting is so far unclear (14–19, 70). For such clinical studies, the MSC source and their characteristics should be clearly defined to induce reproducible responses on T immunosuppression. Such standardization could be potentiated with *in vitro* generated MSCs, and our simple method could therefore be very useful.

To the best of our knowledge, our results represent the first demonstration that immune-modulatory huiPS-MSCs act on human T lymphocytes *in vivo* through a switch from a Th1 inflammatory differentiation pathway to a Treg cell pathway. These findings may promote the development of new strategies, involving pluripotent stem cells and their derived cells, for the induction of specific immune tolerance.

## Ethics Statement

This study was carried out in accordance with the recommendations of our local Institutional Ethic Committee for Laboratory Animals (CIEPAL-Azur, NCE/2013-102), France. The protocol was approved by the C3M animal core facility committee (INSERM U1065, Université de Nice, France).

## Author Contributions

CR: collection and/or assembly of data, data analysis and interpretation, manuscript writing, and final approval of manuscript. GS, JP, and LI: collection and/or assembly of data, data analysis and interpretation, and final approval of manuscript. NB, GD, CV, and AB: collection and/or assembly of data and final approval of manuscript. NM and AW: data analysis and interpretation and final approval of manuscript. CB-W: data analysis and interpretation, financial support, and final approval of manuscript. MR: conception and design, financial support, collection and/or assembly of data, data analysis and interpretation, manuscript writing, and final approval of manuscript.

## Conflict of Interest Statement

The authors declare that the research was conducted in the absence of any commercial or financial relationships that could be construed as a potential conflict of interest.
