# Supplementary material for: Immunosuppressive Mesenchymal Stromal Cells Derived from Human-Induced Pluripotent Stem Cells Induce Human Regulatory T Cells In Vitro and In Vivo
Source: Front Immunol. 2018 Jan 25;8:1991. doi: 10.3389/fimmu.2017.01991 (PMC5788894; doi:10.3389/fimmu.2017.01991)
Supplement: Supplementary file 1 [file Presentation_1.PDF]

Suppl Figure 1

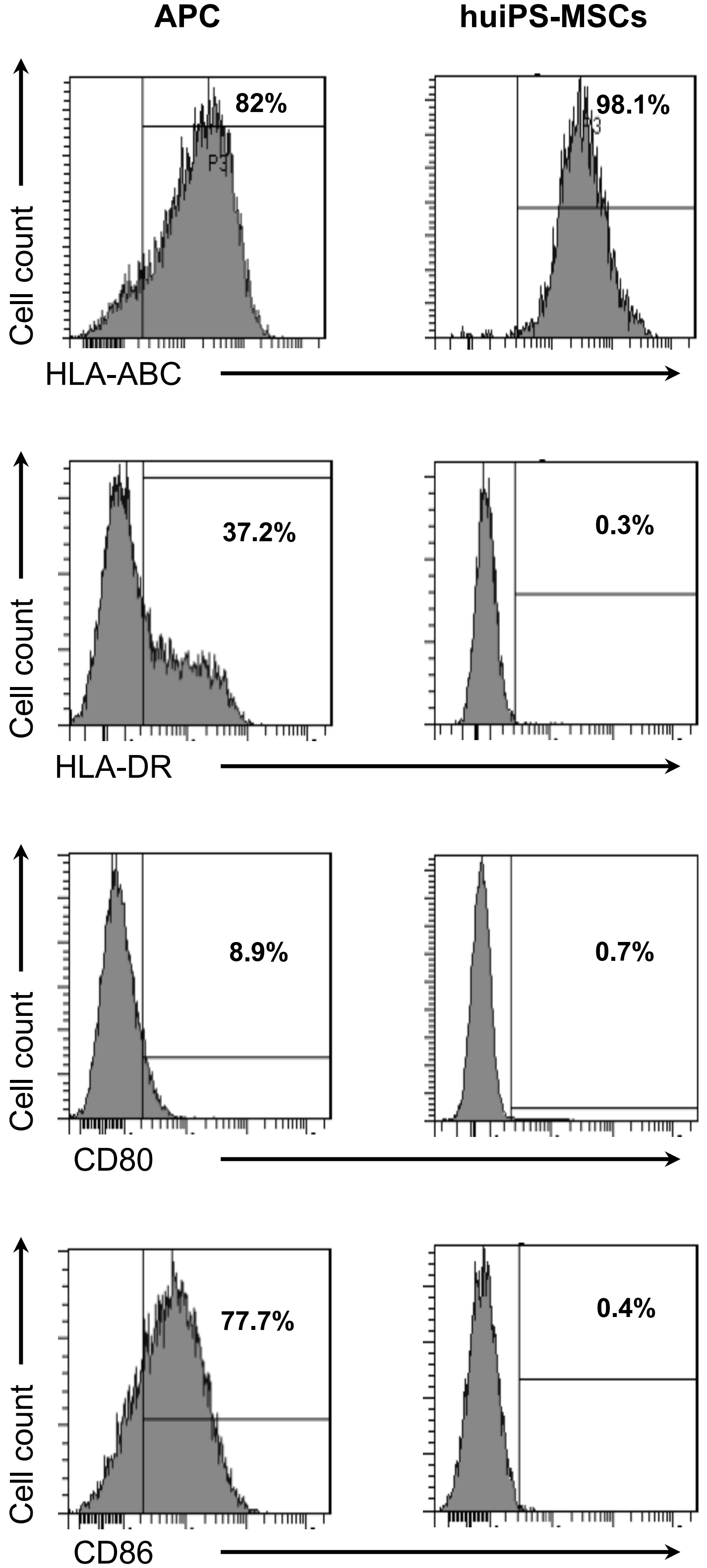

Supplementary figure 1: huIPS-MSCs display the same level of expression of HLA-ABC than control human cells but no expression of HLA-DR nor co-stimulatory CD80 and CD86 molecules. Flow cytometry analysis of huiPS-MSCs for the expression of HLA-ABC, HLA-DR and the co-stimulatory CD80 and CD86 molecules compared to human control APC cells (human activated B lymphocytes). The corresponding % of positive cells are indicated.
