# Supplementary material for: Immunosuppressive Mesenchymal Stromal Cells Derived from Human-Induced Pluripotent Stem Cells Induce Human Regulatory T Cells In Vitro and In Vivo
Source: Front Immunol. 2018 Jan 25;8:1991. doi: 10.3389/fimmu.2017.01991 (PMC5788894; doi:10.3389/fimmu.2017.01991)
Supplement: Supplementary file 2 [file Presentation_2.PDF]

Suppl Fig 2

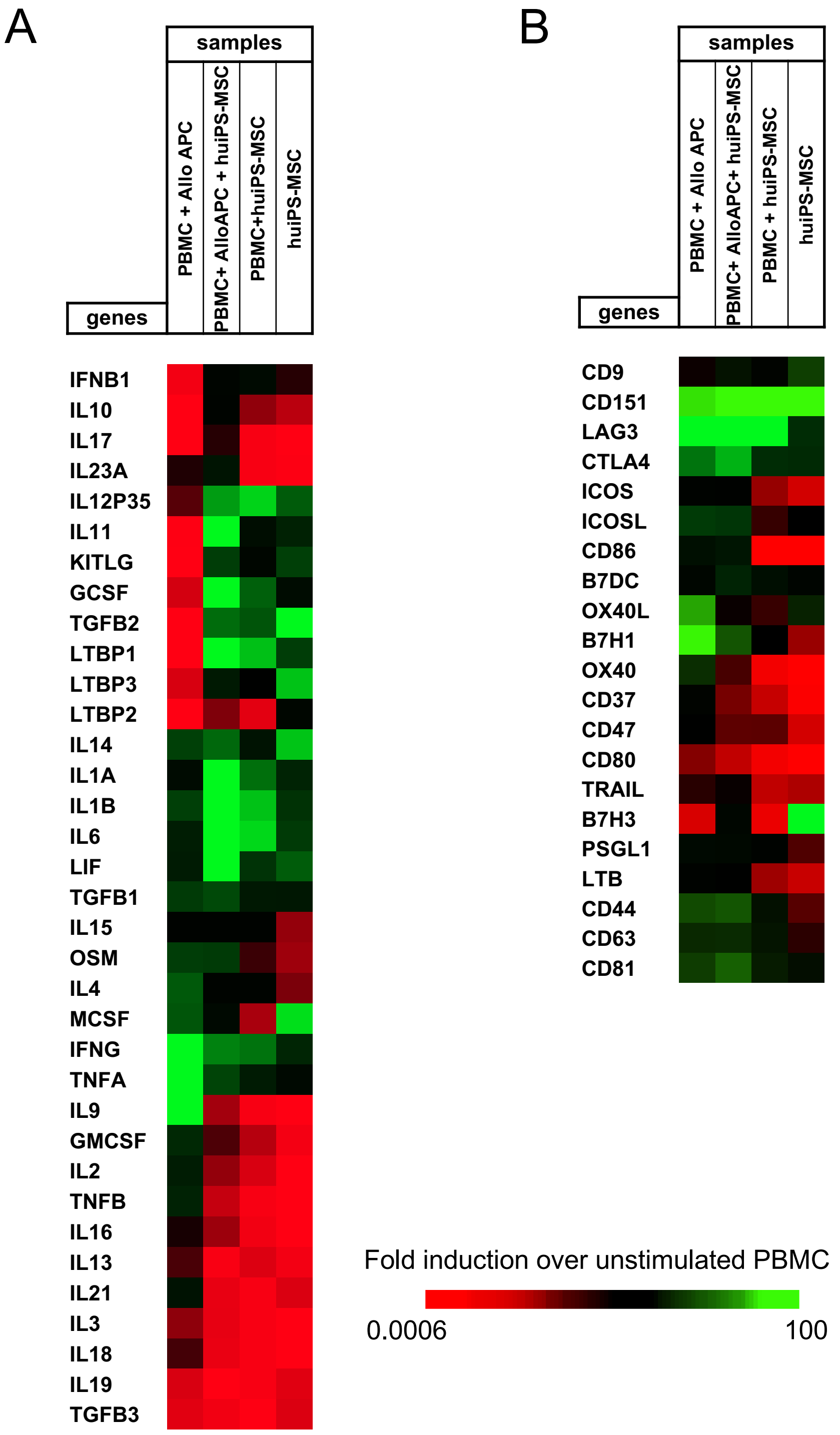

Supplementary figure 2: Selected transcriptomic analysis of MLR of allogenic stimulated T cells in the presence or not of huiPS-MSCs . A real time quantitative RT-PCR analysis was performed on the RNA collected at the onset of an MLR experiment. RNA from unstimulated PBMC, from allogenic stimulated PBMC in the presence or not of huiPS-MSCs, from not stimulated PBMC in the presence of huiPS-MSCs, and from huiPS-MSCs were analyzed. Ct values were normalized to the mean of 3 different housekeeping genes (*ACTIN*, *GAPDH* and *UBIQUITIN*). Differences were calculated with the $2^{-\Delta\Delta Ct}$ method and compared to the relative level of expression detected in unstimulated PBMC and were represented in heat maps. (A) transcript signature of selected cytokines and secreted factors. (B) transcript signature of accessory molecules involved in the T cells activation. The red colors indicate lower expression compared to unstimulated PBMC, the green colors corresponding to a higher expression.
