# Supplementary material for: Immunosuppressive Mesenchymal Stromal Cells Derived from Human-Induced Pluripotent Stem Cells Induce Human Regulatory T Cells In Vitro and In Vivo
Source: Front Immunol. 2018 Jan 25;8:1991. doi: 10.3389/fimmu.2017.01991 (PMC5788894; doi:10.3389/fimmu.2017.01991)
Supplement: Supplementary file 3 [file Presentation_3.PDF]

Suppl Fig 3

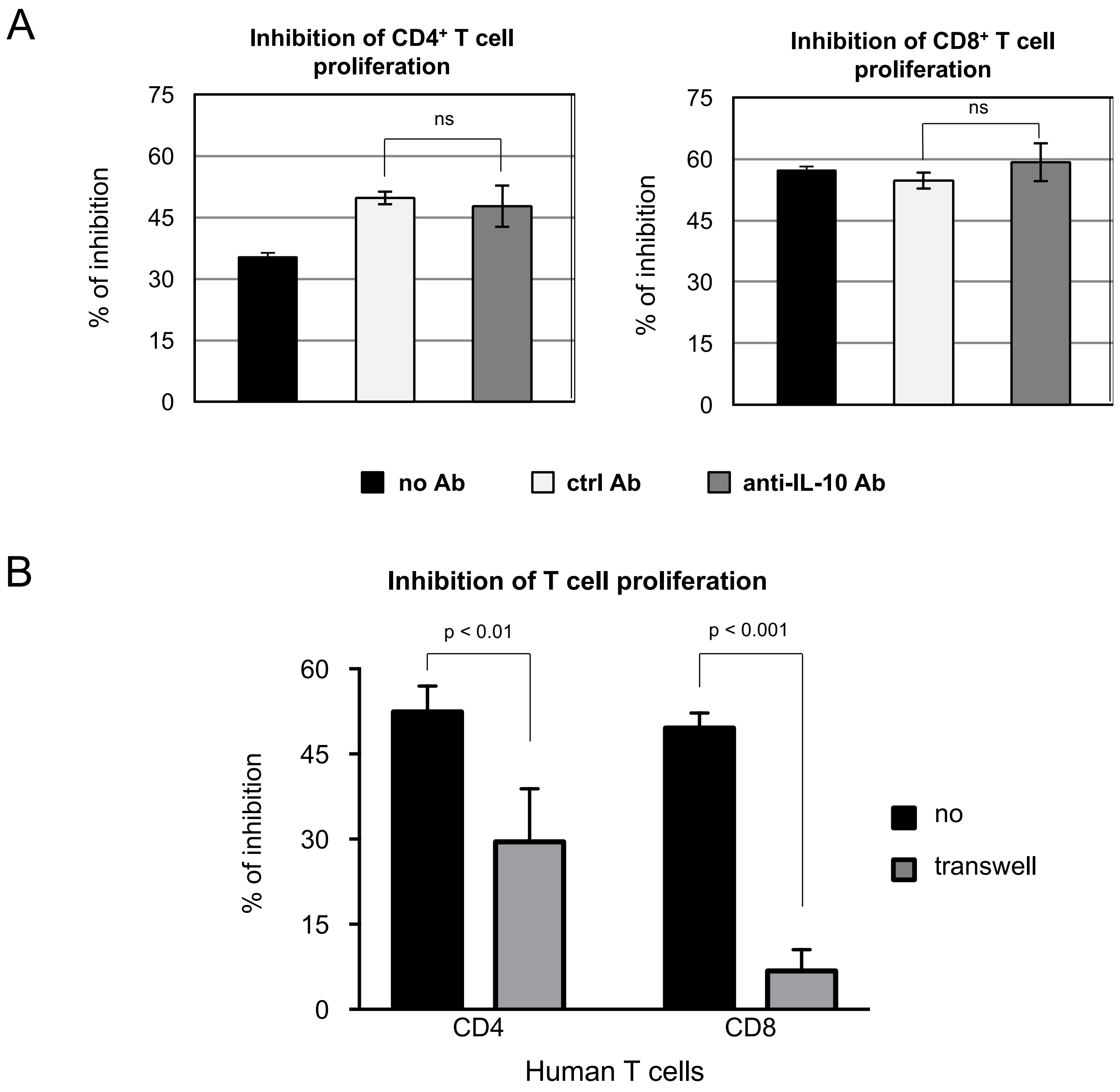

Supplementary figure 3: huIPS-MSCs display an immunosuppressive effect on human T cells proliferation, independent of IL-10 (for both CD4⁺ and CD8⁺ T cells) but partially blocked in transwell assay for CD4⁺ T cells and almost completely for CD8⁺ T cells. (A) Percentage on inhibition of the proliferation of CD4⁺ T cells (left panel) and CD8⁺ T cells (right panel) in MLR with huiPS-MSCs in the absence of blocking antibodies (no Ab) or in the presence of an isotype control antibody (ctrl Ab) or a blocking anti-IL-10 antibody (anti-IL-10 Ab) (both at 10µg/ml concentration). (B) Percentage on inhibition of the proliferation of CD4⁺ T cells (left side) and CD8⁺ T cells (right side) in MLR in the presence of huiPS-MSCs (no, black bars) or in transwell assay (human T cells in an insert separating them from huiPS-MSCs, transwell, gray bars). Both (A) and (B), bars represent the mean ± SD of independent experiments (3 for (A) and 2 for (B)).
