# Supplementary material for: Immunosuppressive Mesenchymal Stromal Cells Derived from Human-Induced Pluripotent Stem Cells Induce Human Regulatory T Cells In Vitro and In Vivo
Source: Front Immunol. 2018 Jan 25;8:1991. doi: 10.3389/fimmu.2017.01991 (PMC5788894; doi:10.3389/fimmu.2017.01991)
Supplement: Supplementary file 4 [file Presentation_4.PDF]

## Suppl Fig 4

A Activated CD4+ T cells in MLR without huiPS-MSC

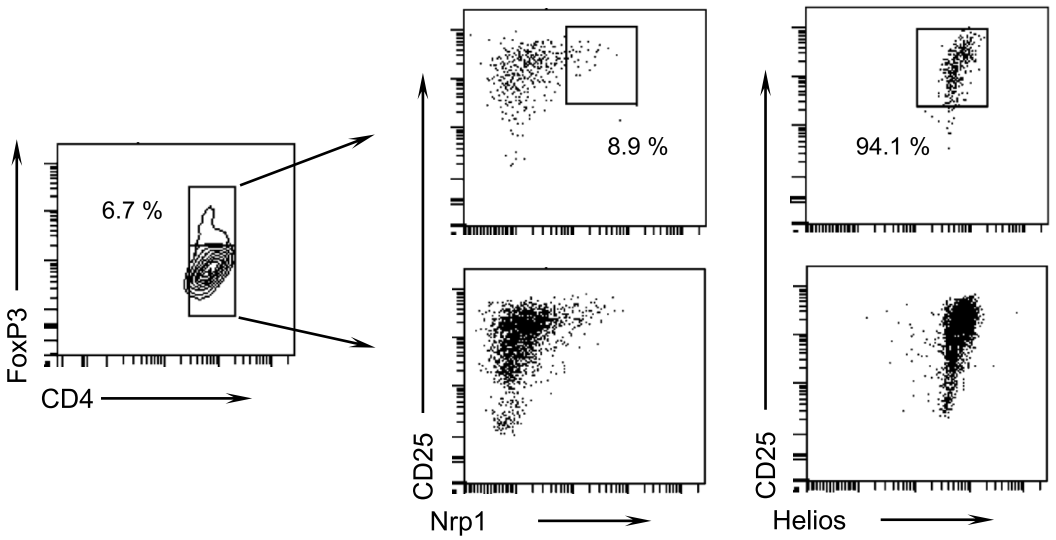


B Activated CD4+ T cells in MLR with huiPS-MSC

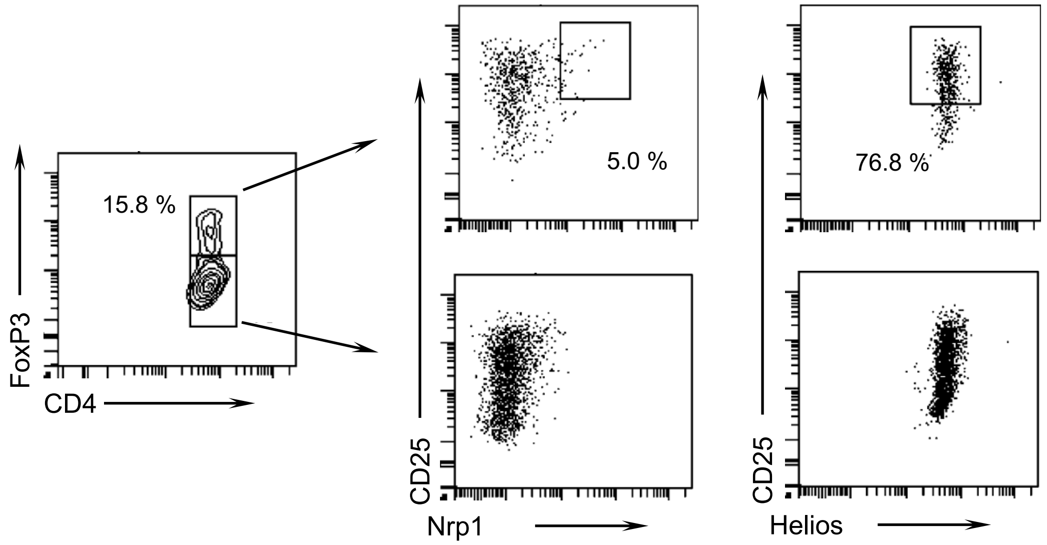


*Supplementary figure 4: Phenotypic characterization of the $CD4^+$ Treg cells generated in MLR with huiPS-MSCs with the Neuropilin1 (Nrp1) and Helios markers.* Flow cytometry analysis of $CD4^+$ T cells recovered after 6 days in from MLR in the absence (A) or the presence (B) of huiPS-MSCs. $FoxP3^+CD4^+$ and $FoxP3^-CD4^+$ T cell populations were tested for the expression of CD25 and Nrp1 (Left panels) or CD25 and Helios (right panels). The % indicate the proportion of double positive T cells for both markers. The figure displays data obtained from 1 representative experiment out of 3.
