# Supplementary material for: Immunosuppressive Mesenchymal Stromal Cells Derived from Human-Induced Pluripotent Stem Cells Induce Human Regulatory T Cells In Vitro and In Vivo
Source: Front Immunol. 2018 Jan 25;8:1991. doi: 10.3389/fimmu.2017.01991 (PMC5788894; doi:10.3389/fimmu.2017.01991)
Supplement: Supplementary file 5 [file Presentation_5.PDF]

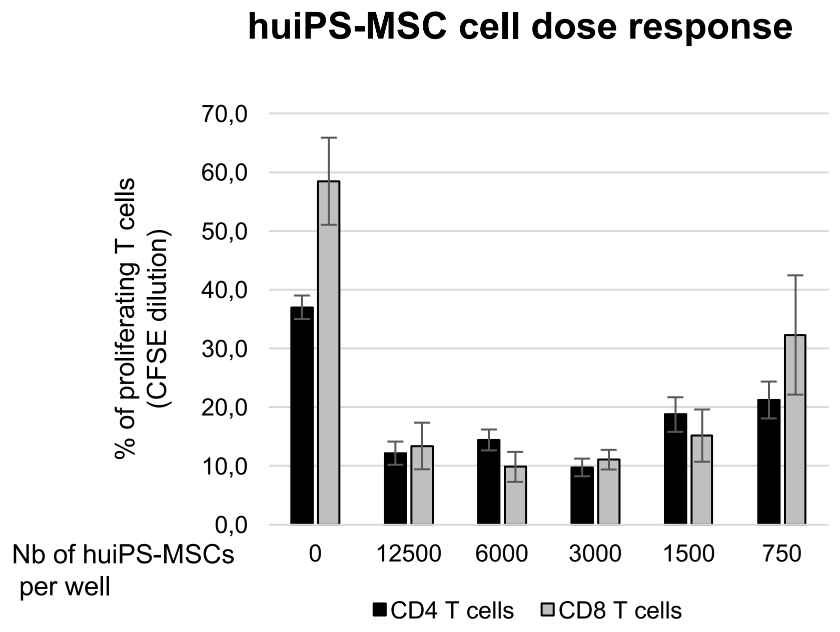

Inhibition of $CD4^+$ and $CD8^+$ T cell proliferation (dilution of CFSE) induced by huiPS-MSCs at different cell number. CFSE stained human PBMC (50 000 cells per well) were stimulated with the same number of stimulatory cells (MLR) in the presence of different amount of huiPS-MSCs ranging from 0 to 12 500 cells per well). The figure displays the mean % of proliferating $CD4^+$ and $CD8^+$ T cells ± SD. Data are from 1 representative experiment.
